# The RZZ complex requires the N-terminus of KNL1 to mediate optimal Mad1 kinetochore localization in human cells

**DOI:** 10.1098/rsob.150160

**Published:** 2015-11-18

**Authors:** Gina V. Caldas, Tina R. Lynch, Ryan Anderson, Sana Afreen, Dileep Varma, Jennifer G. DeLuca

**Affiliations:** 1 Department of Biochemistry and Molecular Biology, Colorado State University, Fort Collins, CO 80523, USA; 2 Natural Resource Ecology Laboratory, Colorado State University, Fort Collins, CO 80523, USA; 3 Department of Cell and Molecular Biology, Feinberg School of Medicine, Northwestern University, Chicago, IL 60611, USA

**Keywords:** kinetochore, Mad1, KNL1, RZZ complex, Bub1, spindle checkpoint

## Abstract

The spindle assembly checkpoint is a surveillance mechanism that blocks anaphase onset until all chromosomes are properly attached to microtubules of the mitotic spindle. Checkpoint activity requires kinetochore localization of Mad1/Mad2 to inhibit activation of the anaphase promoting complex/cyclosome in the presence of unattached kinetochores. In budding yeast and *Caenorhabditis elegans*, Bub1, recruited to kinetochores through KNL1, recruits Mad1/Mad2 by direct linkage with Mad1. However, in human cells it is not yet established which kinetochore protein(s) function as the Mad1/Mad2 receptor. Both Bub1 and the RZZ complex have been implicated in Mad1/Mad2 kinetochore recruitment; however, their specific roles remain unclear. Here, we investigate the contributions of Bub1, RZZ and KNL1 to Mad1/Mad2 kinetochore recruitment. We find that the RZZ complex localizes to the N-terminus of KNL1, downstream of Bub1, to mediate robust Mad1/Mad2 kinetochore localization. Our data also point to the existence of a KNL1-, Bub1-independent mechanism for RZZ and Mad1/Mad2 kinetochore recruitment. Based on our results, we propose that in humans, the primary mediator for Mad1/Mad2 kinetochore localization is the RZZ complex.

## 1. Introduction

The spindle assembly checkpoint (SAC) senses attachment between kinetochores and the mitotic spindle, and prevents anaphase until all kinetochores are properly connected to spindle microtubules (MTs). Checkpoint signalling critically depends on the recruitment of Mad1 and Mad2 to kinetochores. How Mad1/Mad2 is recruited to kinetochores to activate the SAC is still unclear; however, a series of evolutionarily conserved dependencies have been established. The kinetochore protein KNL1 has emerged as a primary scaffold for checkpoint complexes at kinetochores [1–3]. Phosphorylated Met-Glu-Leu-Thr (MELT) motifs in the middle and N-terminal regions of KNL1 directly bind to a complex of Bub3/Bub1, which is required for localization of Mad1/Mad2 [4–8]. The protein Zwint1, which binds the C-terminal region of KNL1, is proposed to recruit the RZZ complex, which is also implicated in Mad1 kinetochore recruitment [9–12]. Together, this evidence suggests that KNL1 mediates Mad1 recruitment in a bimodal manner, through its N-terminal region via Bub1/Bub3 and through its C-terminal region via RZZ/Zwint1 [4–6,13–16]. Recently, however, super-resolution mapping of protein localization at kinetochores demonstrated that RZZ complex components localize near the KNL1 N-terminus, at a region spatially distinct from Zwint1 [17]. In addition, kinetochore localization of RZZ depends on Bub1 in human cells [18]. Thus, the mechanism by which Bub1 and the RZZ complex contribute to Mad1 kinetochore localization, and the potential link between these two recruitment pathways, are still unclear. Here, we demonstrate that the RZZ complex requires the N-terminal region of KNL1 independent of kinetochore-localized Zwint1 but dependent on Bub1 to mediate robust Mad1 localization in human cells. In addition, we find evidence for a KNL1-independent RZZ recruitment pathway that, although likely to contribute minimally to kinetochore RZZ levels, provides an alternative route for Mad1/Mad2 localization and checkpoint functionality. In contrast to previously described models [19,20], our data suggest that the RZZ complex serves as the main mediator for Mad1 kinetochore recruitment in human cells.

## 2. Results and discussion

### 2.1. Both Bub1 and the RZZ complex are required for optimal Mad1 kinetochore recruitment

**Figure 1.**
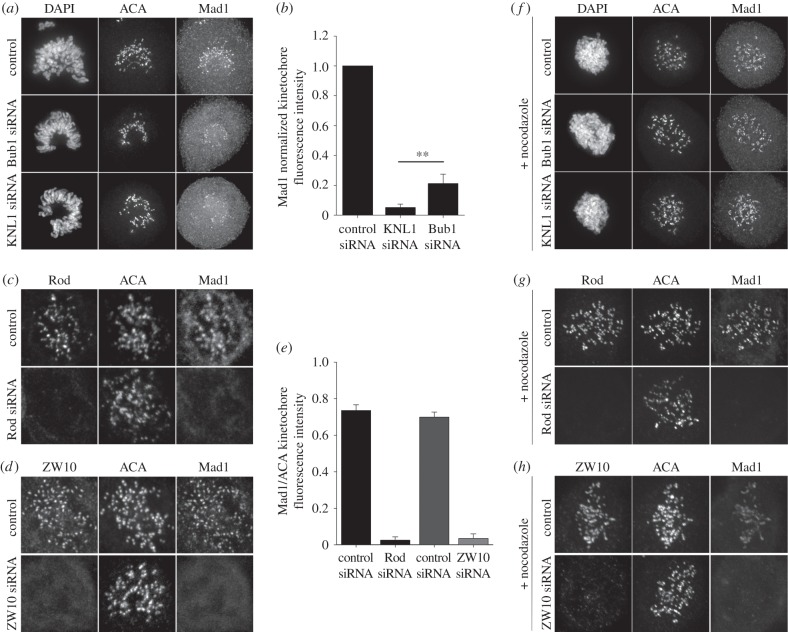
The RZZ complex is critical for Mad1 kinetochore localization. HeLa cells depleted of (*a*,*f*, middle row) Bub1, (*a*,*f*, bottom row) KNL1, (*c*,*g*) Rod and (*d*,*h*) ZW10 were stained for ACA, Mad1 and (*a*,*f*) DAPI, (*c*,*g*) Rod or (*d*,*h*) ZW10, as indicated. The controls for each experiment are shown in the top rows for each panel, and the nocodazole-treated samples are shown in panels (*f*–*h*) (all rows). (*b*,*e*) Normalized Mad1 kinetochore fluorescence intensity was measured in control cells and those treated with the respective siRNAs as indicated in the bar graph. Error bars represent s.d. between experiments. (*b*) *n* > 182 kinetochores, *n* > 7 cells per experiment, *n* = 3 independent experiments, **p < 0.05 (Student's *t*-test). (*e*) *n* = 76 kinetochores, *n* = 3 cells per experiment, *n* = 2 independent experiments for Rod siRNA, *n* = 58 kinetochores, *n* = 3 cells per experiment and *n* = 2 independent experiments for ZW10 siRNA.

To test whether Bub1 and RZZ differentially contribute to Mad1 localization, we depleted HeLa cells of KNL1, Bub1 or the RZZ components Rod and ZW10 independently, and quantified the levels of Mad1 at kinetochores by immunofluorescence (figure 1; electronic supplementary material, figure S1). Depletion of KNL1, Rod or ZW10 resulted in minimal levels of Mad1 at kinetochores (<5% of control levels), while Bub1 depletion resulted in a more modest reduction in kinetochore-associated Mad1 (approx. 20% of control levels; figure 1*a–e*). We do not believe that the Mad1 remaining at kinetochores after Bub1 RNAi is due to incomplete Bub1 depletion since Bub1 was not detected at kinetochores by immunofluorescence in these cells co-stained for Mad1 and Bub1, and treatment of Bub1-depleted cells with nocodazole does not result in accumulation of detectable kinetochore-associated Bub1 [15] (electronic supplementary material, figure S1). Therefore, we conclude that Bub1 and RZZ contribute differentially to Mad1 kinetochore recruitment. To further test this idea, we examined the levels of kinetochore Mad1 after KNL1, Bub1 or RZZ depletion in the presence of the MT depolymerizing drug nocodazole. In agreement with our previous result, we observed significant levels of kinetochore Mad1 in Bub1-depleted cells, but no detectable Mad1 after RZZ depletion and nocodazole treatment (figure 1*f–h*). Interestingly, we also observed kinetochore Mad1 in KNL1-depleted cells in the presence of nocodazole (figure 1*f*; electronic supplementary material, figure S1). These results demonstrate that Mad1 can be recruited at low levels to kinetochores (compared with control) in the absence of Bub1 and, surprisingly, in the absence of KNL1. We propose that both Bub1 and the RZZ complex are required for robust Mad1 kinetochore recruitment, but, while recruitment via Bub1 is dispensable for low levels of Mad1 localization, recruitment via RZZ is necessary for the localization of Mad1 to kinetochores, even at low levels.

### 2.2. KNL1 N-terminus is necessary for kinetochore accumulation of the RZZ complex

**Figure 2.**
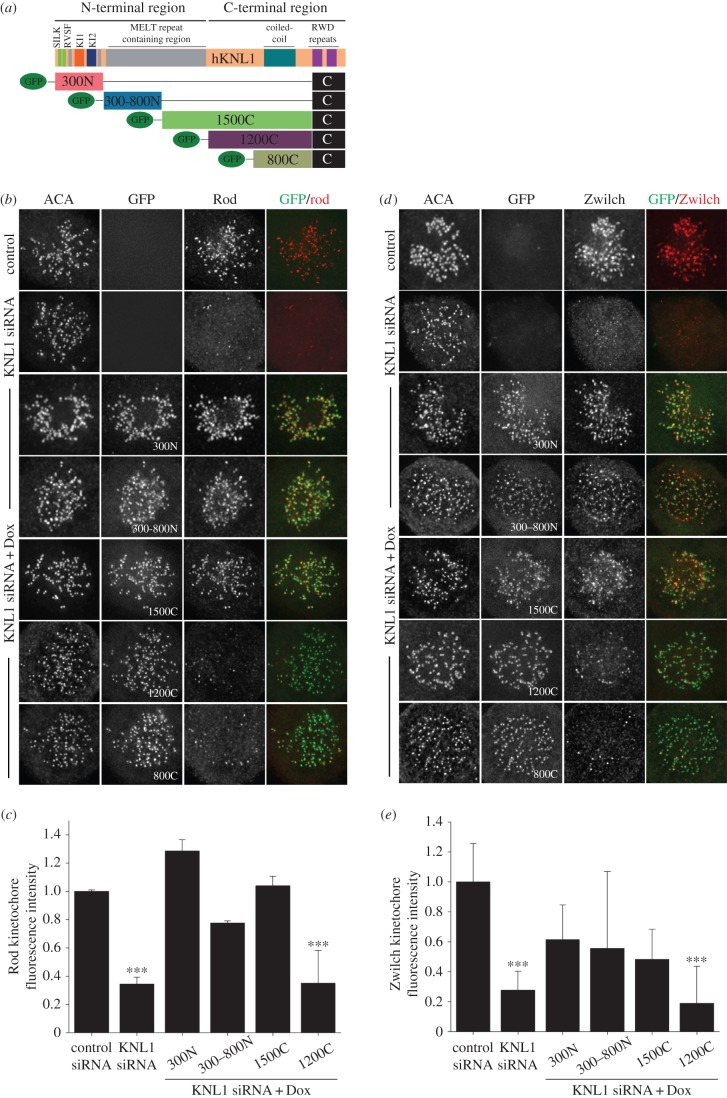
KNL-1 N-terminus is required for RZZ kinetochore localization. (*a*) Schematic of GFP-KNL1 constructs stably incorporated into Flp-In T-REx HeLa cell lines [15]; the amino acids of KNL1 constructs shown are as follows: amino acids 1–300 (300N), 300–818 (300–800N), 1174–2316 (1200C) 1519–2316 (800C), 819–2316 (1500C), 2056–2316. (*b*,*d*) Flp-In T-REx HeLa cells were depleted of endogenous KNL1, rescued with the indicated GFP-KNL1 fragment upon doxycycline addition and immunostained with the indicated antibody. (*c*,*e*) Kinetochore fluorescence intensities were measured in control cells, KNL1-depleted cells and GFP-KNL1 Flp-In T-REx cells treated with KNL1 siRNA and doxycycline. Error bars represent s.e.m. (*c*) *n* > 720 kinetochores, *n* > 12 cells per condition and *n* = 2 independent experiments. (*e*) *n* > 240 kinetochores and *n* > 4 cells per condition. (*c*,*e*) ***p < 0.001 (Mann–Whitney rank sum test).

We and others have demonstrated that KNL1 is required for Bub1 localization to kinetochores [2,3,15,21,22]. However, recruitment of the RZZ complex after KNL1 depletion has not been extensively examined, and significant differences in the amount of remaining RZZ in the absence of KNL1 have been reported [17,18]. We find that after KNL1 depletion, kinetochore localization of RZZ components Rod, Zwilch and ZW10 is reduced to approximately 30% of control levels (figure 2*b–e*; electronic supplementary material, figure S2). This suggests that the absence of KNL1 does not completely abolish RZZ recruitment, and explains our observation that Mad1 binds kinetochores in the absence of KNL1 and in the presence of nocodazole (figure 1*f*). A recent study revealed an interaction between the NDC80 complex and the RZZ complex component Rod in *C. elegans* [23]. Although this interaction has not been described in human cells, depletion of the human NDC80 component Hec1 results in decreased RZZ kinetochore localization [17,24]. Thus, an alternative RZZ kinetochore recruitment pathway, relying on NDC80, could function in humans. In line with this premise, the remaining NDC80 at kinetochores after KNL1 depletion (approx. 60%) [15] may explain the partial retention of kinetochore RZZ upon KNL1 depletion we find in this study. It still needs to be determined, however, whether reduction of RZZ after NDC80 depletion in human cells is caused by decreased Mps1-mediated phosphorylation of KNL1.

The N-terminal half of KNL1, which is enriched for MELT motifs, is required for Bub1 kinetochore localization [3,15,21,22]. In addition, KNL1 is required for the kinetochore localization of the RZZ complex, but the key domains required for this function have not been determined. Previous studies demonstrated an interaction between the ZW10 subunit of RZZ and Zwint1, a protein that binds to the C-terminus of KNL1, and is considered central to RZZ kinetochore recruitment [10,13,14]. More recent studies, however, demonstrated that components of the RZZ complex physically map to a region near the KNL1 N-terminus [17]. We hence asked which region of KNL1 was important for recruitment of RZZ by carrying out immunofluorescence rescue experiments using HeLa cells inducibly expressing a series of GFP-KNL1 fragments (figure 2). Cells expressing N-terminal KNL1 fragments 300N, 300–800N and 1500C (all of which contain a minimal C-terminal kinetochore binding domain) were sufficient to drive kinetochore accumulation of the three RZZ components, Rod, Zwilch and ZW10; whereas cells expressing C-terminal KNL1 fragments 1200C and 800C did not facilitate robust RZZ kinetochore recruitment (figure 2*b–e*; electronic supplementary material, figure S2). By contrast, only cells expressing the C-terminal KNL1 fragments 1500C, 1200C and 800C, which contain the predicted Zwint1 binding region (aa 1834–2107), facilitated Zwint1 recruitment (electronic supplementary material, figure S2). As for RZZ, identical localization requirements were observed for the protein Spindly, which is recruited to kinetochores through the RZZ complex (electronic supplementary material, figure S3). Whether the previously demonstrated direct interaction between ZW10 and Zwint1 [10,13], either at kinetochores or in the cytoplasm, is required for RZZ localization to the KNL1 N-terminus remains to be tested. Regardless, these results demonstrate that kinetochore-bound Zwint1 is not necessary or sufficient for the recruitment of RZZ and that the KNL1 N-terminus contributes significantly to RZZ kinetochore localization. While preparing this manuscript, a study was published demonstrating that a KNL1 fragment containing four MELT repeats, competent to recruit Bub1 to kinetochores, was also able to mediate ZW10 kinetochore localization [18]. Our results are consistent with these findings regarding ZW10 (electronic supplementary material, figure S2) and further demonstrate that MELT domain-containing KNL1 fragments are similarly required for Zwilch, Rod and Spindly kinetochore localization (figure 2*a*,*e*). Our results, together with the recent data from Zhang *et al*. [18], support a model in which formation of a complex between Bub1 and RZZ components is necessary for optimal Mad1 localization to kinetochores.

### 2.3. KNL1 N-terminus is necessary for kinetochore accumulation of Mad1/Mad2

**Figure 3.**
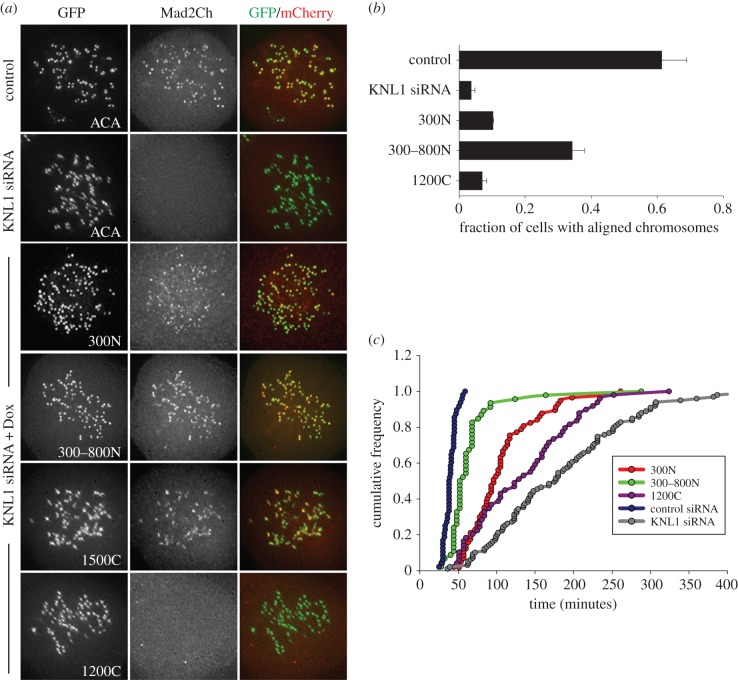
KNL1 N-terminus is required for Mad2 kinetochore localization. (*a*–*c*) Flp-In T-REx HeLa cells were depleted of endogenous KNL1, rescued with the indicated GFP-KNL1 fragment upon doxycycline addition, and (*a*) overexpressed for Mad2-mCherry, (*b*) analysed for chromosome alignment or (*c*) analysed by live-cell imaging to determine time from nuclear envelope breakdown to anaphase. (*b*) Error bars represent s.d. between experiments, *n* = 3 independent experiments and *n* ≥ 50 cells scored per experiment. (*c*) Cumulative frequency of timing through mitosis.

Because the N-terminal half of KNL1 mediates recruitment of both Bub1 and RZZ components, which are required for Mad1 kinetochore localization, we predicted that the KNL1 N-terminus is necessary for Mad2 recruitment. We examined the localization of mCherry-tagged Mad2 in our GFP-KNL1-expressing cell lines (figure 3*a*). As predicted, Mad2 localized to kinetochores in cells expressing 300N, 300–800N and 1500C KNL1, and did not localize to kinetochores in cells expressing 1200C KNL1 (figure 3*a*). We recently demonstrated that the N-terminal region of KNL1 is necessary and sufficient for Aurora B-mediated regulation of kinetochore-MT attachments [15]. Because the N-terminal region of KNL1 is able to promote both Aurora B activity and accumulation of checkpoint components, we asked whether the KNL1 N-terminus was sufficient for chromosome alignment and timely mitotic progression. In agreement with Vleugel *et al*. [2], depletion of KNL1 caused severe chromosome misalignment (figure 3b). A C-terminal KNL1 fragment (1200C) was not able to recover alignment defects, and cells expressing 300–800N KNL1 partially restored alignment defects (figure 3b) [2]. Also in agreement with Vleugel *et al*., 300N KNL1 (similar to 250N used in their study [2]) did not fully restore alignment defects, possibly because 300N KNL1 does not fully rescue Aurora B-mediated phosphorylation of kinetochore substrates [2,15]. We next analysed the timing through mitosis in cells expressing various KNL1 fragments. Consistent with our chromosome alignment data, cells expressing 300N KNL1 exhibited a long delay in mitosis, while cells expressing 300–800N KNL1 exhibited only a slight delay (figure 3c). Interestingly, KNL1-depleted cells and 1200C-expressing cells also exhibited a long mitotic delay despite their inability to accumulate high levels of Mad2. Thus, our data indicate that under these conditions, mitotic timing correlates with the cell's ability to align its chromosomes, despite high or low levels of Mad1/Mad2 at kinetochores, and that low levels of Mad1/Mad2 in the absence of KNL1 or the KNL1 N-terminus are sufficient to maintain a functional checkpoint. Indeed, KNL1-depleted cells arrested in mitosis in the presence of nocodazole for at least 6 h (data not shown). Our results are in agreement with previous studies demonstrating that only when KNL1-depleted cells or cells expressing KNL1 C-terminal fragments are challenged with nocodazole and small amounts of reversine (an Mps1 inhibitor) is the checkpoint abrogated [2]. However, we note that these results contrast with other studies demonstrating that depletion of KNL1 does, in fact, result in checkpoint abrogation [25,26]. Together, our data demonstrate that the KNL1 N-terminus is necessary for high levels of kinetochore Mad1/Mad2 localization and support the existence of a KNL1-independent Mad1/Mad2 recruitment pathway.

### 2.4. Bub1 is required for kinetochore recruitment of the RZZ complex

**Figure 4.**
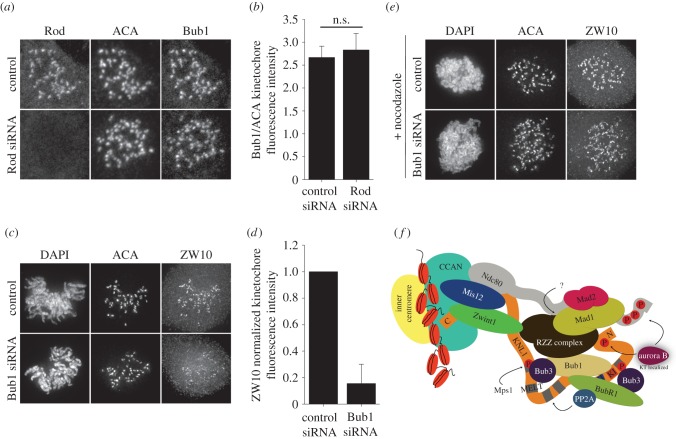
The RZZ complex requires Bub1 for proper kinetochore localization. (*a*–*d*) HeLa cells were depleted of Rod or Bub1, and immunostained for ACA and either Bub1 or the RZZ component ZW10. (*b*,*d*) Kinetochore fluorescence intensities were measured in control cells and those treated with the respective siRNAs as indicated in the bar graph. Error bars represent s.d. (*b*) *n* = 59 kinetochores, *n* = 3 cells per experiment, *n* = 2 independent experiments and n.s. = not statistically significantly different. (*d*) *n* > 192 kinetochores, *n* > 12 cells per experiment and *n* = 2 independent experiments. (*e*) HeLa cells depleted of Bub1 were treated with nocodazole, fixed, and immunostained for ACA and ZW10. (*f*) Model of Mad1/Mad2 kinetochore recruitment. KNL1-dependent recruitment of the RZZ complex to kinetochores is mediated by the KNL1 N-terminus and Bub1. Together, the RZZ complex and Bub1 mediate recruitment of Mad1 and Mad2. Whether the RZZ complex directly interacts with Mad1 is unknown. This model proposes a KNL1-independent mechanism for RZZ/Mad1 recruitment, possibly through the NDC80 complex.

Our results indicate that the RZZ complex relies on the KNL1 N-terminus for optimal kinetochore localization. The N-terminus of KNL1 is necessary for Bub1 recruitment, and Bub1 is also required for optimal Mad1 kinetochore localization. Therefore, we predicted some level of co-dependency between RZZ and Bub1 for their kinetochore localization. Depletion of the RZZ component Rod resulted in similar levels of kinetochore-localized Bub1 to those observed in control cells (figure 4*a*,*b*), whereas depletion of Bub1 significantly reduced the levels of ZW10 at kinetochores, suggesting that Bub1 is necessary for wild-type levels of the RZZ complex at kinetochores (figure 4*c*,*d*). This is in agreement with a recent study demonstrating dependence of RZZ kinetochore localization on the central region of Bub1 [18]. Based on these results, we postulate that Bub1 and the RZZ complex interact at the N-terminal region of KNL1 to mediate Mad1 kinetochore recruitment.

Similar to our results after KNL1 depletion, treatment of Bub1-depleted cells with nocodazole resulted in significant retention of kinetochore-localized ZW10 (figure 4*e*), supporting the idea that the RZZ complex can bind to kinetochores, at low levels, in a KNL1- and Bub1-independent manner. This KNL1-independent mechanism for RZZ kinetochore recruitment, although probably providing a minimal contribution for Mad1 kinetochore binding, could impact checkpoint efficiency significantly upon conditions where checkpoint signalling is needed at its maximum.

### 2.5. Conclusion

Previous studies have implicated two pathways in the kinetochore recruitment of the checkpoint protein Mad1: one mediated by Bub1 binding to the N-terminal region of KNL1 and another by the RZZ complex binding to Zwint1. Whether these two Mad1 recruitment pathways intersected was unclear. We find that both Bub1 and the RZZ complex are required for robust accumulation of Mad1 at kinetochores in human cells. In contrast to the model in which Zwint1 mediates recruitment of the RZZ complex, presumably through KNL1 C-terminus, to facilitate Mad1 localization, we find that the RZZ complex requires the KNL1 N-terminal region independent of Zwint1 kinetochore recruitment. In addition to and in agreement with a recent study [18], we find that Bub1 is required for optimal accumulation of RZZ components. Finally, our results suggest the existence of an RZZ binding site on kinetochores that is independent of Bub1 and KNL1 localization. We propose a model in which RZZ, stabilized by Bub1 and the N-terminus of KNL1, serves as the primary mediator for Mad1 localization (figure 4f). To date, there is no evidence for direct interaction between RZZ components and Mad1/Mad2, Bub1 or KNL1. Thus, *in vitro* binding assays and structural studies will be required to determine the nature of the interactions occurring at the N-terminus of KNL1, and the mechanism by which KNL1 modulates interactions with checkpoint activating and checkpoint silencing proteins to control mitotic progression.

## 3. Material and methods

### 3.1. Cell culture and transfections

HeLa cells (ATCC) and FlpIn T-REx HeLa cells stably expressing GFP-tagged KNL1 fragments were cultured in DMEM (Life Technologies) supplemented with 10% FBS and 1% penicillin/streptomycin at 37°C in 5% CO_2_. For silence and rescue experiments, stable cell lines were doubly blocked with thymidine, depleted of endogenous KNL1 using the corresponding siRNAs and induced with doxycycline for overexpression as described by Caldas *et al*. [15]. siRNA transfections were performed using Oligofectamine (Invitrogen), according to the manufacturer's instructions, and analysed 48 h post-transfection. DNA transfections were performed using Effectene (Qiagen) according to the manufacturer's instructions. Mad2-mCherry DNA transfections were performed 6–7 h after the first KNL1 siRNA transfection and prior to the first thymidine block. For nocodazole experiments, following the second thymidine block, cells were released for 9 h in fresh medium and treated for 1 h with 10 µM nocodazole prior to fixation. For asynchronous cultures, nocodazole was added for 45 min prior to cell fixation. siRNAs targeting KNL1 were 5′-GAACACAUUGCUUUCUGCUCCCAUU-3′, 5′-GGGCAGGAUGACAUGGAGAUCACUA-3′ and 5′-AAGAUCUGAUUAAGGAUCCACGAAA-3′ [25]. Bub1 siRNA was 5′-CAGCUUGUGAUAAAGAGUCAA-3′ purchased from Qiagen. The SMART pool ON-TARGET plus siRNAs used for Rod silencing were 5′-GGAGCUAGCCCUAAGAUUU-3′, 5′-CUCAAGAGAUGCUGAAUUA-3′, 5′-GAUAAAGCAUGGCAGAAUU-3′ and 5′-GUAAAUAACUUGCGAGAGU-3′ (Thermo Scientific). siRNA sequence 5′-AAGGGUGAGGUGUGCAAUAUG-3′, used for ZW10 RNAi, was purchased from Thermo Scientific [27]. Generation of the FlpIn HeLa stable cell lines containing GFP-tagged KNL1 fragments was described by Caldas *et al*. [15] and the fragments are as follows: amino acids 1–300 (300N), 300–818 (300–800N), 1174–2316 (1200C), 1519–2316 (800C) and 819–2316 (1500C). 1500C differs from the sequence published by Bolanos-Garcia *et al*. [28] in that aa 910–1120 are not contained.

### 3.2. Plasmids

Mad2 was cloned into an mCherry-C1 plasmid to generate mCherry-Mad2. The plasmid was purified using an Endo-free Maxi kit (QIAGEN) before transfection.

### 3.3. Immunofluorescence

Fixation of HeLa cells were performed as described previously [29]. In brief, cells were rinsed in 37°C PHEM buffer (60 mM Pipes, 25 mM Hepes, 10 mM EGTA and 4 mM MgSO_4_, pH 6.9), fixed in 4% paraformaldehyde for 20 min and extracted in PHEM buffer + 0.5% Triton X-100 for 5 min. Immunostaining was performed using the following antibodies: rabbit polyclonal anti-Zwint1 (Gene-Tex) was used at 1 : 200; rabbit polyclonal anti-Zwilch, and anti-Mad1 mouse monoclonal (a generous gift from A. Musacchio, Max Planck Institute of Molecular Physiology, Dortmund, Germany), were used at 1 : 200 and 1 : 50 respectively; rabbit polyclonal anti-ZW10 (a generous gift from R. Vallee, Columbia University, New York, NY, USA), was used at 1 : 200; rabbit polyclonal anti-Rod (a generous gift from G. Chan, University of Alberta, Edmonton, Alberta, Canada), was used at 1 : 200; human anti-centromere antibody (ACA; Antibodies, Inc.) was used at 1 : 500; mouse anti-Bub1 (EMD Millipore) was used at 1 : 500; and rabbit polyclonal anti-Mad1 (Genetex) was used at 1 : 500. Secondary antibodies conjugated to Alexa 488, Alexa 647 (Life Technologies) or Rhodamine Red-X (Jackson ImmunoResearch Laboratories, Inc.) were used at 1 : 300.

### 3.4. Image acquisition and analysis

For image acquisition, three-dimensional stacks were obtained through the cell using either a DeltaVision Personal DV microscope (Applied Precision) equipped with a CoolSNAP HQ2 Camera (Photometrics/Roper Scientific), a 60 ×/1.42 NA PlanApochromat objective (Olympus), and SoftWoRx acquisition software (Applied Precision) or a Nikon Eclipse TiE inverted microscope equipped with a Yokogawa CSUX1 spinning disc, an Andor iXion Ultra888 EM-CCD camera and a 100 ×/1.4 NA (Planapo) DIC oil immersion objective (Nikon). For fixed cell experiments, images were acquired at room temperature as Z-stacks at 0.2 µm intervals. Kinetochore fluorescence intensity measurements were performed using MetaMorph software (Molecular Devices). The integrated fluorescence intensity minus the calculated background was determined for each kinetochore in control and treated samples, and normalized to the average value obtained from control cells [30]. For quantifications shown in figure 2, images were converted into maximal intensity projections and kinetochore fluorescence intensity quantification was performed with a custom script using the image processing SciKit (skimage) package in Python. A Gaussian filter was applied for background reduction, and Otsu's method was used to calculate a clustering-based image threshold for automated kinetochore identification. Background values for each image were calculated using the averaged pixel value of the cell and subsequently removed from the extracted kinetochore intensity values.

### 3.5. Live-cell imaging

HeLa cells in 35 mm glass-bottomed dishes (MatTek Corporation) were transfected with KNL1 siRNA and induced with doxycycline as described above. Cells were imaged in Leibovitz's L-15 media (Invitrogen) supplemented with 10% FBS, 7 mM Hepes, pH 7.0 and 4.5 g l^−1^ glucose. Stage temperature was maintained at 37°C with an environmental chamber (Precision Control). Fluorescence images of GFP-KNL1-expressing cells were acquired every 5 min for 7–8 h.

## Supplementary Material

Depletion of KNL1 and Bub1 from HeLa cells. Domain requirements of KNL1 for kinetochore localization of ZW10 and Zwint1. Domain requirements of KNL1 for kinetochore localization of Spindly
