# Supplementary material for: The RZZ complex requires the N-terminus of KNL1 to mediate optimal Mad1 kinetochore localization in human cells
Source: Open Biol. 2015 Nov 18;5(11):150160. doi: 10.1098/rsob.150160 (PMC4680571; doi:10.1098/rsob.150160)

Supplementary Figures

Supplementary Fig. S1. Depletion of KNL1 and Bub1 from HeLa cells. (a) HeLa cells were depleted of KNL1 or Bub1, fixed, and immunostained for ACA and Bub1. (b) HeLa cells depleted of KNL1 or Bub1 were treated with nocodazole, fixed, and immunostained for ACA and Bub1. (c) Percentage of HeLa cells with detectable levels of KNL1 after KNL1 depletion and KNL1 immunostaining relative to control.

Supplementary Fig. S2. Domain requirements of KNL1 for kinetochore localization of ZW10 and Zwint1. (a,b) Flp-In T-REx HeLa cells were depleted of endogenous KNL1, rescued with the indicated GFP-KNL1 fragment upon doxycycline addition and immunostained with an antibody against ZW10 (a) or Zwint1 (b).

Supplementary Fig. S3. Domain requirements of KNL1 for kinetochore localization of Spindly. Flp-In T-REx HeLa cells were depleted of endogenous KNL1, rescued with the indicated GFP-KNL1 fragment upon doxycycline addition and immunostained with an antibody against Spindly.

SUPPLEMENTARY FIGURE S1

(a)

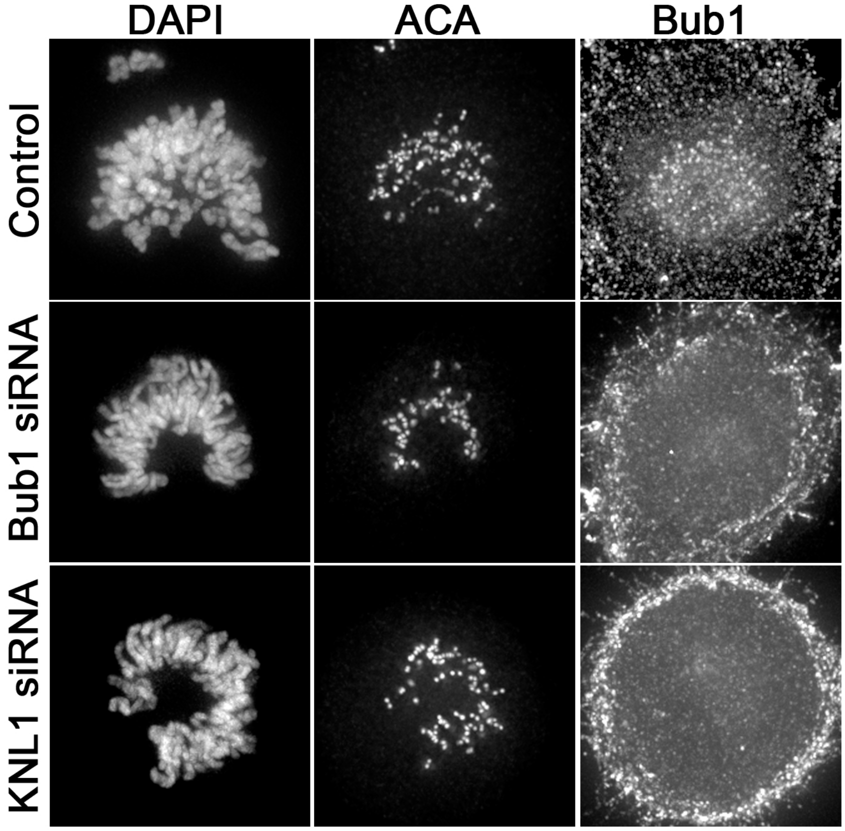

(b)

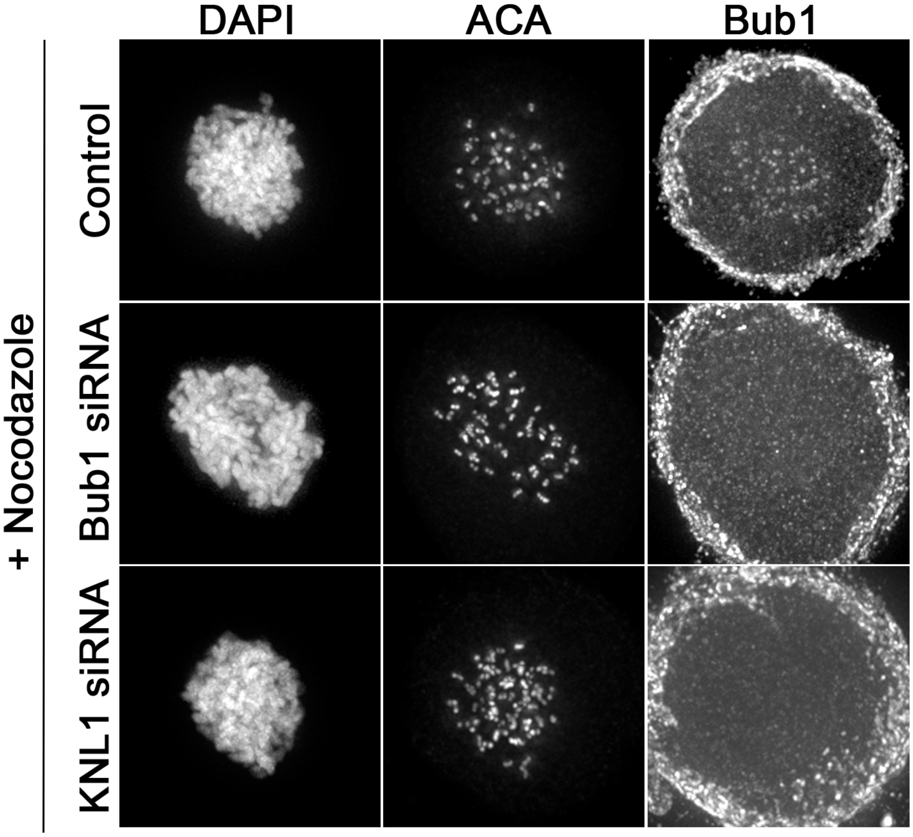

(c)

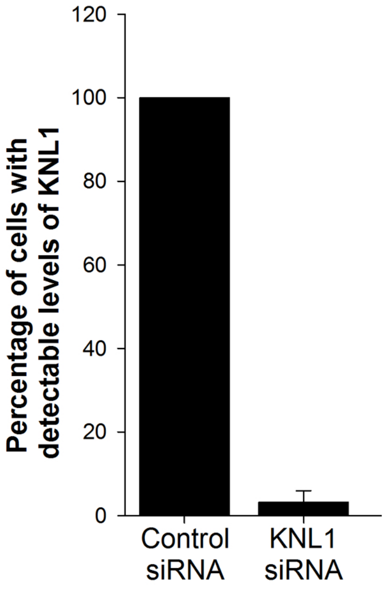

SUPPLEMENTARY FIGURE S2

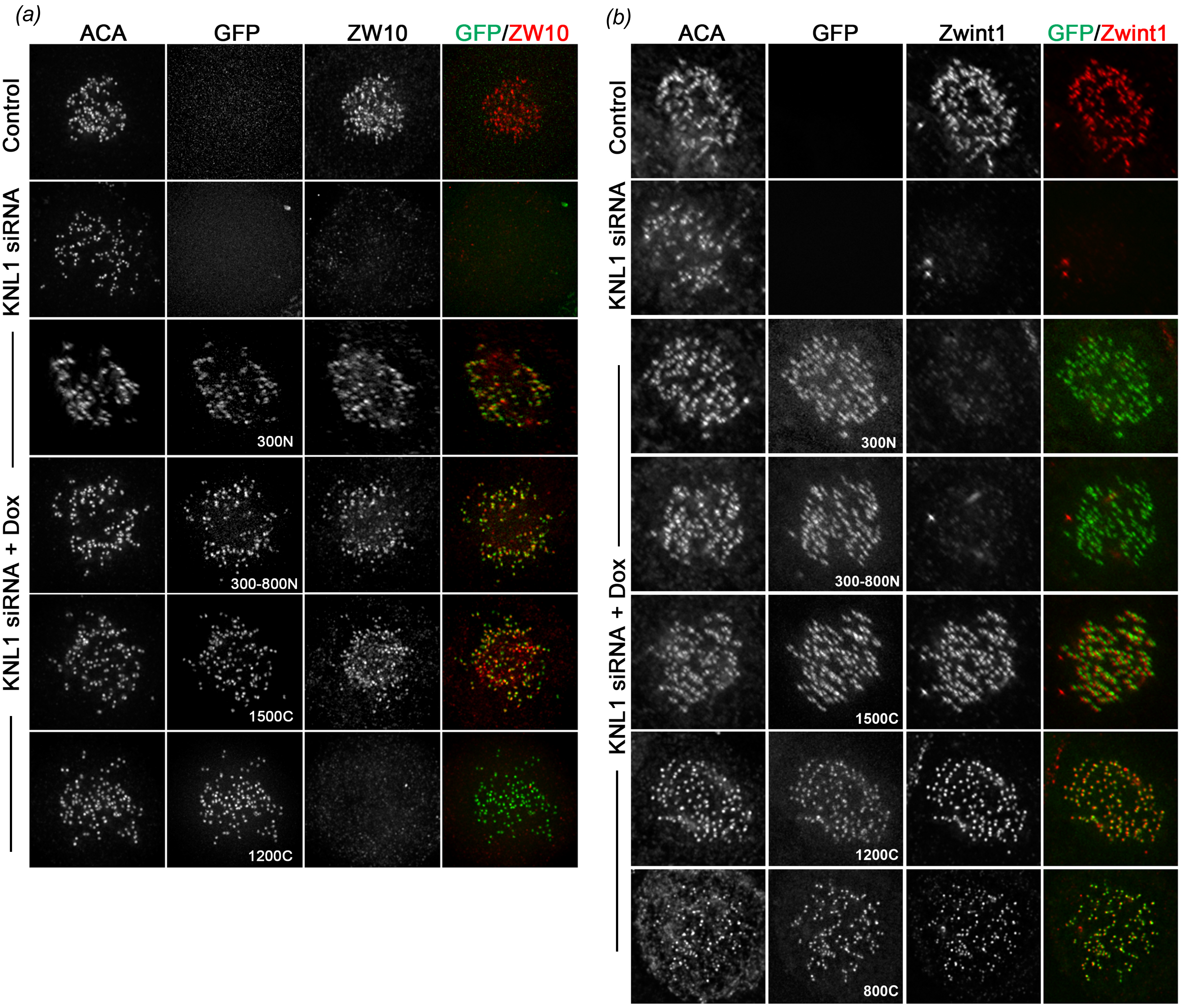

SUPPLEMENTARY FIGURE S3

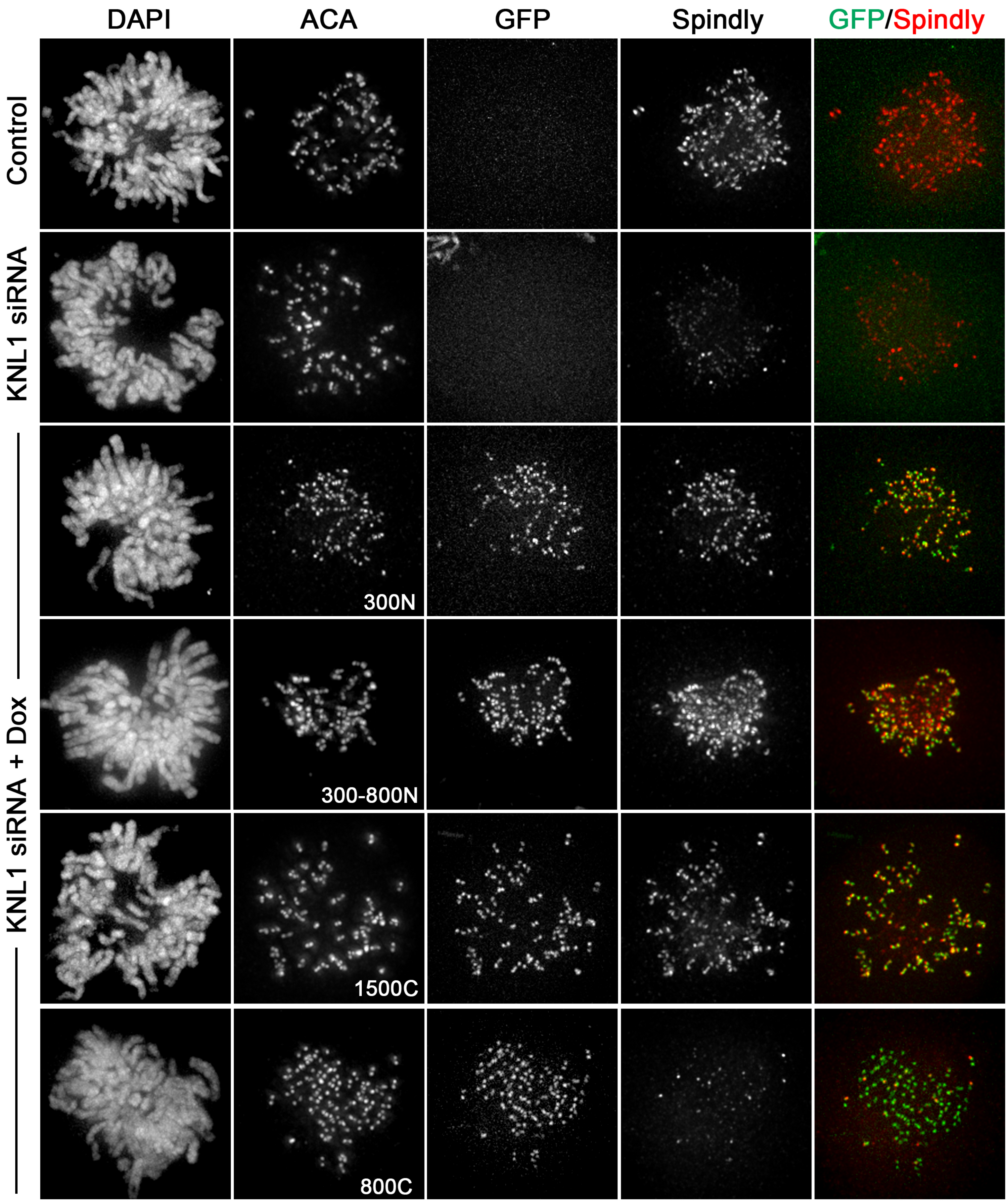

Supplement: Depletion of KNL1 and Bub1 from HeLa cells. Domain requirements of KNL1 for kinetochore localization of ZW10 and Zwint1. Domain requirements of KNL1 for kinetochore localization of Spindly [file rsob150160supp1.pdf]
